# Factors Influencing Resonance Frequency Analysis (RFA) Measurements and 5-Year Survival of Neoss Dental Implants

**DOI:** 10.1155/2019/3209872

**Published:** 2019-04-01

**Authors:** Peter Andersson, Luca Pagliani, Damiano Verrocchi, Stefano Volpe, Herman Sahlin, Lars Sennerby

**Affiliations:** ^1^Private Practice, Clinica Feltre, Viale 14 Agosto 1866, No. 31, 32032 Feltre, Italy; ^2^Private Practice, Via Giuseppe Mercalli 11, Milano, Italy; ^3^Private Practice, Via Angelo Guadagnini 21, 38054 Fiera Di Primiero, Italy; ^4^Private Practice, Piazza del Fante 10, 00195 Rome, Italy; ^5^Neoss AB, Arvid Wallgrens Backe 20, 413 46 Gothenburg, Sweden; ^6^Department of Oral & Maxillofacial Surgery, Institute of Odontology, Sahlgrenska Academy, University of Gothenburg, P.O. Box 450, 405 30 Gothenburg, Sweden

## Abstract

**Background:**

Diagnostic instruments based on resonance frequency analysis (RFA) can be utilised to assess dental implant stability during treatment and follow-up.

**Aim:**

The aim of the present study was to investigate the influence of patient- and implant-related factors on implant stability and the 5-year implant survival. In addition, the influence of stability (ISQ value) at placement and abutment connection on implant survival was evaluated.

**Materials and Methods:**

RFA measurements from a total of 334 consecutive patients with 745 dental implants (Neoss Ltd., Harrogate, UK) were retrospectively analysed after at least 5 years in function. Statistics were used to evaluate the influence of the different variables on implant stability and implant survival. Odds ratio calculations were performed to compare the risk for implant failure using 60, 65, 70, and 75 ISQ as threshold levels at placement and loading.

**Results:**

A total of 20 implant failures in 14 patients were noted during the 5 years of follow-up, giving an overall cumulative survival rate (CSR) of 97.3% at the implant level and 95.8% at the patient level. Gender, jaw, position, bone quality, and implant diameter had an influence on implant stability at placement. Jaw, bone quality, and implant diameter had an influence on stability after 3-4 months of healing. More failures were observed in full than in partial rehabilitations. Age, gender, jaw, position, bone quantity, bone quality, implant diameter, and implant length had no influence on implant survival. Implants with ISQ values below the threshold levels showed lower survival rates compared to implants with values above these levels.

**Conclusions:**

The present study showed a significantly higher risk for implant failure, showing an ISQ value below 70 and 75 at placement or after 3-4 months of healing. The results indicate that RFA measurements can be used to identify implants with increased risk for failure.

## 1. Introduction

Firm primary implant stability is regarded as a determinant of success in implant dentistry [1]. This notion is based on early observations that implant failure is more common in the presence of compromising biomechanical factors, such as in case of low bone density, short implants, grafted bone, premature loading, and overloading [2, 3]. One explanation is that low implant stability may lead to micromotions at the bone-implant interface during healing and loading, which in turn stimulates to fibrous soft tissue formation rather than bone healing. Diagnostic instruments based on resonance frequency analysis (RFA) are available for assessment of implant stability as a function of interface stiffness during treatment and follow-up [4]. The technique measures the resonance frequency (RF) of a transducer that is attached to the implant. With the existing commercial instruments, the RF in Hz is translated to an implant stability quotient (ISQ) unit between 1 and 100, where increased values reflect increased stability [5]. Experimental research using displacement measurements has shown a correlation between RFA and micromobility when applying a lateral load to the dental implant [6]. The bone density at the implant site seems to be the most important determinant of primary stability [6–9], although factors such as implant design, length, and diameter seem to affect RFA measurements as well [10, 11]. Experimental and clinical studies have demonstrated an increase of ISQ units with time as a consequence of bone formation and maturation at the implant interface [12–14]. According to the manufacturers and some authors, the technique can be used to measure that sufficient stability has been achieved prior to as well as identifying implants with low stability in order to take measures to avoid implant failure [4]. Although clinical studies have shown implants with low and/or falling ISQ units over time to be more prone to failure [10, 15, 16], the ability of the RFA technique to predict implant failure has been questioned by several authors [17–19].

The aim of the present study was to retrospectively investigate the influence of patient- (age, gender, jaw, indication, bone density, and bone volume) and implant-related (diameter and length) factors on implant stability as assessed by RFA measurements and the 5-year implant survival in 334 consecutive implant patients. In addition, the influence of stability (ISQ value) at placement and abutment connection on implant survival was evaluated.

## 2. Materials and Methods

### 2.1. Patients

Data related to patient, type of treatment, implants, bone conditions, and outcomes at annual check-ups were extracted from a simple computerised system (MS Excel, Microsoft, Redmond, USA) used to keep track on consecutive implant treatments at the three centres participating in the study. A total of 334 consecutive patients (55.4% female, 44.6% male, mean age 52 ± 11.8 years) who met the following inclusion criterion were included in the present retrospective analysis: implant treatment for replacement of one or several teeth using a one- or two-stage procedure with 3 to 4 months of healing to prosthetic loading and follow-up for at least 5 years in function (Table 1). Immediate/early loading cases were not included. All patients had been given their written consent to the treatment plan and follow-up according to the routine procedures at the centres. The study followed the directives given by the Ethical Committee at the Feltre Hospital, Feltre, Italy, and in accordance with the World Medical Association Declaration of Helsinki.

**Figure 1:**
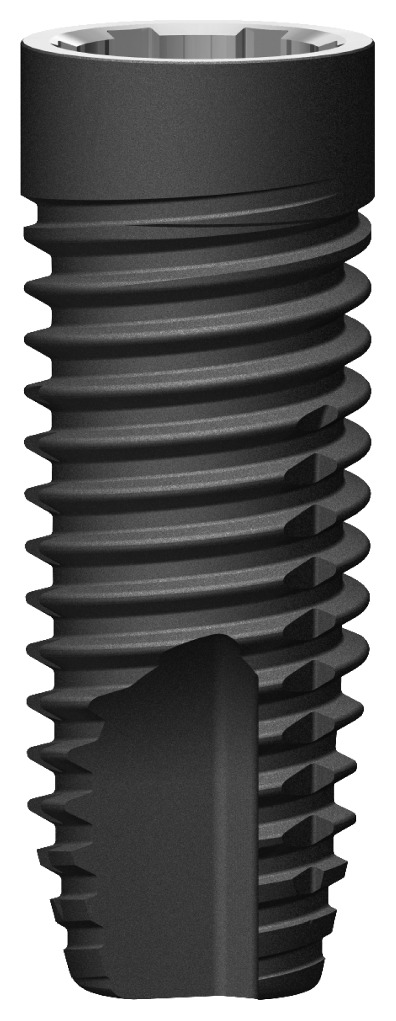
The design of the Neoss implant used in the study.

**Figure 2:**
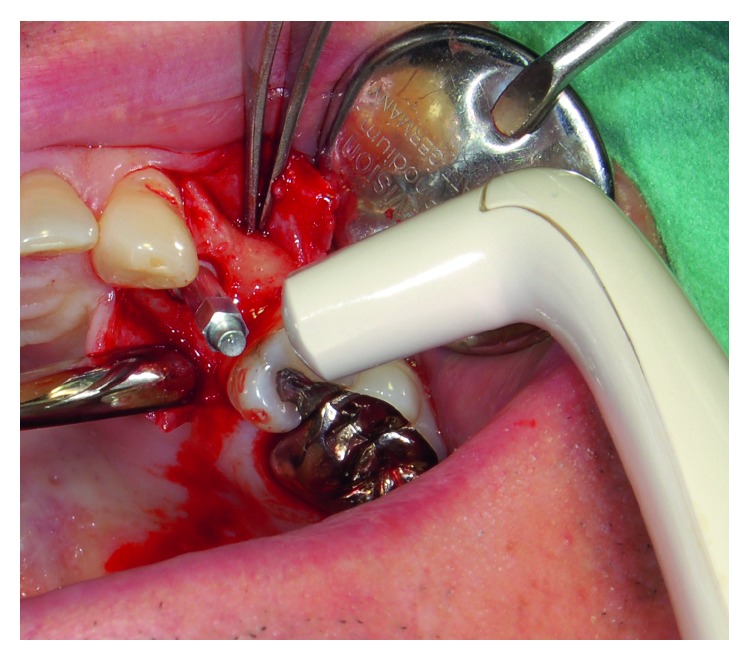
RFA measurement of an implant utilising a SmartPeg™ transducer and an Osstell Mentor™ machine (Osstell AB, Gothenburg, Sweden).

The patients had received 745 dental implants (Neoss Implant system, Neoss Ltd., Harrogate, UK) in diameters from 3.5 to 5.5 mm and in lengths from 7 to 17 mm in all tooth positions in both jaws (Figure 1, Table 2). The implant has a slightly tapered geometry (one degree) and a modified surface obtained by double particle blasting (Bimodal surface). The roughness is higher on the body and less on the neck. The implant system uses an internal flat-to-flat connection with the prosthetic parts. Bone quantity and quality had been evaluated according to the Lekholm and Zarb index [20] (Table 3). The majority of implants had been measured with RFA at placement (*n* = 655) and at abutment connection or prosthetic loading (*n* = 529) 3–6 months after implant placement by using an Osstell Mentor™ machine (Osstell AB, Gothenburg, Sweden) in implant stability quotient values (ISQ) (Figure 2).

The implants were supporting 165 single crowns, 180 fixed partial bridges, 19 fixed full bridges, and 16 overdentures (Tables 4 and 5). The prostheses were made on Neolink™ abutments (Neoss Ltd., Harrogate, UK) made of titanium or gold depending on the material of the framework. Both porcelain and acrylic teeth were used in the study. At two of the centres, most prostheses were screw-retained and the access holes covered with composite fillings. Individual titanium abutments for cementation were also used in some cases to correct implant angulation. At the third centre, only cemented prosthetics had been used.

The patients had been attending annual check-ups and any adverse events such as implant failure, soft tissue problems, or technical complications were noted in the patient charts. In this study, only implant failure was used as a parameter.

### 2.2. Variables and Statistics

The Pearson chi-Squared and Fischer's exact test were used to evaluate the influence of the different variables on implant stability and implant survival. A statistically significant difference was regarded if *p* ≤ 0.05. Odds ratio calculations were performed to compare the risk for implant failure with 60, 65, 70, and 75 ISQ as threshold values at placement or loading.

The variables tested were age, gender, jaw, indication (single tooth, partial jaw, and full jaw), bone quantity, bone quality, implant diameter, and implant length. In addition, the influence of the ISQ value at placement and abutment connection on implant survival was evaluated.

## 3. Results

Five-year survival data could be extracted from 328 of the 334 patients. Six patients with 13 implants had dropped out because of death (*n* = 2) or they had moved (*n* = 4).

A total of 20 implant failures in 14 patients were noted during the 5 years of follow-up giving an overall cumulative survival rate (CSR) of 97.3% at the implant level and 95.8% at the patient level. Eight (1.1%) were early failures (before one year of loading), and 12 (1.6%) were late failures (after one year of loading) (Table 6).

Analyses of RFA measurements at implant placement showed an influence of gender (higher ISQ in males, *p*=0.002), jaw (higher ISQ in mandibles, *p*=0.001), position (*p*=0.037), bone quality (*p*=0.001), and implant diameter (*p*=0.034) (Table 7). Analyses of RFA measurements from abutment connection or prosthetic loading revealed an influence of jaw (*p*=0.001), bone quality (*p*=0.025), and implant diameter (*p*=0.009) (Table 8).

Statistical analyses of the influence of different factors on implant survival showed more failures in full than in partial rehabilitations (*p*=0.003). Age, gender, jaw, position, bone quantity, bone quality, implant diameter, and implant length had no influence on implant survival (Table 9).

**Figure 3:**
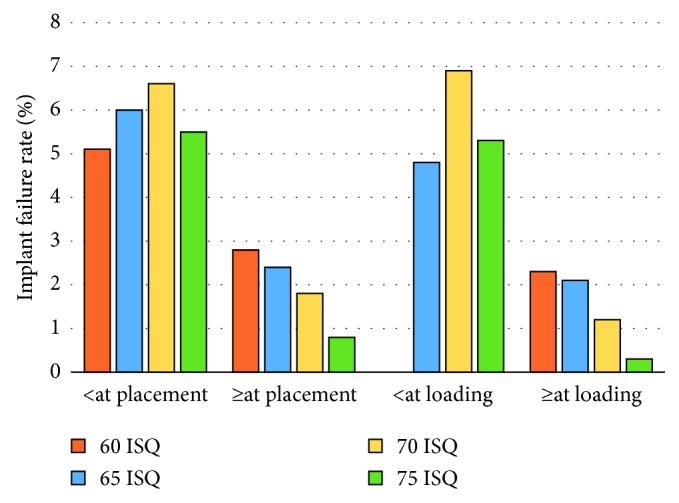
Failure rates for implants with values < or ≥60, 65, 70, and 75 ISQ at placement and loading. *Note.* There is a consistent correlation between failure and stability for implants above the threshold levels at placement and loading (see Table 10 for statistical analyses).

In general, implants with ISQ values below the threshold levels of 60, 65, 70, and 75 ISQ at placement and loading showed lower survival rates compared to implants with values above these levels (Table 10, Figure 3). Moreover, the failure rate decreased with increasing threshold level for implants with ISQ levels ≥ the threshold, while this was not observed for implants below the threshold levels (Figure 3). The differences were statistically significant for threshold values of 70 and 75 ISQ at both placement and loading. When analysing ISQ values from implant placement surgery, the risk for implants failure increased to 6.3 times (*p*=0.003) and 17.9 times (*p*=0.001) for implants below 70 and 75 ISQ, respectively (Table 10).

## 4. Discussion

The present retrospective study showed good clinical outcome with the Neoss implant system after at least five years of loading. The CSR of 97.3 % corresponds well to the 5-year results from other studies on the same [21] and other modern implant systems [22]. In an analysis of data from the Swedish Social Insurance Agency, Derks et al. reported a nine-year survival rate of 97.0% for 2367 implants of eight different brands placed in 596 patients [23].

The analysis of factors influencing the RFA measurements confirmed in large what have been found and reviewed in other publications [4, 5]. There was a relation between bone density and ISQ values, which in turn explains the difference seen between the maxilla and the mandible. Implant length had no influence on the stability in the present study, which is in line with some studies [24–26], although other authors have found a correlation [10]. However, 4.5 mm wide implants were significantly more stable than 4.0 mm ones, which corroborates with the study by Östman et al. [27]. Studies have demonstrated a correlation between marginal bone thickness and ISQ measurements [28–30], which can explain the lack of influence of implant length and the positive effect of implant diameter in the present study. Interestingly, significantly lower ISQ values were found in women than in men. This has also been noted in other studies [11, 31, 32] and may be related to the higher incidence of osteoporosis in women.

Clinical follow-up studies on the first generation of dental implants with a machined surface have shown higher failure rates for implants placed in compromised bone situations [1]. This includes short implants placed in small bone volumes [33] as well as implants in sites with low bone density [2, 3]. In the present study using a surface-modified implant, 207 of the 745 implants were 7 mm or 9 mm long and no difference in failure rate compared to the longer implants was found. Neither did bone density had an effect on implant failure. This is in line with studies on other modern implants [33] and underlines the importance of implant surface modification for good integration [34]. Reviews of the literature have shown improved overall outcomes with surface modified implants compared with machined surfaced implants and particularly in compromised bone situations [35–37].

Interestingly, implants with low stability showed lower survival rates than implants with high stability when using 60, 65, 70, and 75 ISQ as threshold levels at placement. The similar was seen at loading but with one exception. Moreover, it was clear that the failure rate for implants above each threshold level decreased in a linear fashion with increased threshold levels. However, the opposite, i.e., more failures with decreasing threshold values for implants below these levels, could not be observed. In fact, significant differences were only seen for the 70 and 75 ISQ threshold levels. The lack of differences for the 60 and 65 ISQ threshold levels is explained by the fact that too few values were available in the groups with lower values, which hampered the statistical analyses. Moreover, it is possible that some failures were due to factors not related to stability, such as infection. Nevertheless, the risk for implant failure was 3.9 times higher with ISQ values below than above 70 at implant placement and 6.9 times higher when using 75 ISQ as a threshold value. This is in line with the idea that implant micromotion increases the risk for soft tissue integration during initial healing, which is the reason most implant surgeons strive for high primary implant stability [38]. Moreover, it is logical that implants with weak bone integration at prosthesis connection pose a higher risk for failure than well-integrated implants, which was shown by the 6.3 times higher risk for implants with values below 70 ISQ at abutment/prosthesis connection and 17.9 times higher risk when using 75 ISQ as threshold value. Our data corroborate with the outcomes of other studies showing an increased risk for implant failure with decreased stability [10, 15, 16]. For instance, in a retrospective study on 300 implants of which 20 were lost after three years, Turkyilmaz and McGlumphy found a significant difference between failed and successful implants when comparing the primary stabilities (46.5 ± 4 ISQ vs 67.1 ± 7 ISQ). Rodrigo et al. evaluated 37 failures of 542 implants after three years of follow-up and found no correlation between primary stability and implant failure but a significant association when measurements were made after a mean period of 2.8 months after placement. This is in line with Atieh et al. who concluded that implant stability measurements after 8 weeks showed a better accuracy in predicting implants that were at risk of failure than those taken at the time of implant placement [39].

The indication for implant treatment influenced the 5-year implant survival rate in the present study, i.e., more failures were observed in full jaw than in partial jaw cases. The majority of failures, i.e., seven of eight failures in the full jaw rehabilitations were seen in the maxilla. Both these observations are in line with previous experiences from dental implants [1] and are probably related to morphological differences between the jaws [40]. The RFA analysis showed lower stability for maxillary than for mandibular implants as also observed by other authors [7, 27]. Moreover, the ISQ values were lower in full than in partial and single cases. Since the main determinant of RFA is bone density [6, 9], these results are in part explained by the fact that soft bone densities are more commonly found in the maxilla than in the mandible. Moreover, it may also be so that implants placed for single or partial prostheses are protected against overload by the remaining dentition, while full jaw constructions have to take the full load during healing and loading with an increased risk for implant failure.

In conclusion, a 5-year CSR of 97.3% was found in the present patient group. More failures were observed in full than in partial rehabilitations. Age, gender, jaw, position, bone quantity, bone quality, implant diameter, and implant length had no influence on implant survival. Gender, jaw, position, bone quality, and implant diameter had an influence on implant stability at placement. Jaw, bone quality, and implant diameter had an influence on stability after 3-4 months of healing. In general, the present study also showed lower survival rates for implants with low stability than for implants with higher stability at the different threshold levels. There was a significantly higher risk for failure for implants with an ISQ value below 70 and 75 than for implants with higher stability at placement. Moreover, the risk for failure increased further if the ISQ value was still below 70 and 75 after 3-4 months of healing. Since implants with high stability seem to be more successful, it can be speculated that measures to improve implant stability may improve the clinical outcome. However, clinical studies are needed to evaluate the impact of such measures, such as adapted drilling protocols, the use of wider and tapered implants, and prolonged healing periods, on implant stability and implant survival.

## Figures and Tables

**Table 1:** Number of patients and implants according to gender.

	Patients		Implants	
	*n*	%	*n*	%
Female	183	55.5	434	59.0
Male	147	44.5	301	41.0
Total	330		735	

Information is missing for 4 patients with 10 implants.

**Table 2:** Implant lengths and diameters.

		Diameter					Total
		3.5 mm	4.0 mm	4.5 mm	5.0 mm	5.5 mm	
Length	7 mm	5	16	10	0	0	31
	9 mm	27	98	41	2	8	176
	11 mm	37	118	42	0	5	202
	13 mm	49	126	20	1	2	198
	15 mm	33	86	9	0	0	128
	17 mm	4	3	0	0	0	7
Total		155	447	122	3	15	742

**Table 3:** Bone quantity and quality according to Ga and Albrektsson [20].

		Bone quantity					
		A	B	C	D	E	Total
Bone quality	1	0	8	22	7	0	37
	2	7	184	191	10	0	392
	3	23	104	127	9	0	263
	4	0	24	25	1	0	50
Total		30	320	365	27	0	742

Information is missing for 3 implants.

**Table 4:** Type of prosthetic constructions.

	Maxilla		Mandible		Total	
	*n*	%	*n*	%	*n*	%
Single	86	46.2	79	40.7	165	43.4
Partial	86	46.2	94	48.5	180	47.4
Full arch, fixed	14	7.5	5	2.6	19	5.0
Full arch, overdenture	0	0	16	8.2	16	4.2
Total	186		194		380	

**Table 5:** Number of implants per type of construction.

	Maxilla		Mandible		Total	
	*n*	%	*n*	%	*n*	%
Single	86	23.4	79	21.0	165	22.2
Partial	212	57.8	240	63.8	452	60.8
Full	69	18.8	57	15.2	126	17.0
Total	367		376		743^*∗*^	

^*∗*^Information is missing for 2 implants.

**Table 6:** Implant survival.

Interval	Implants	Failed	Withdrawn	CSR (%)
Insertion to loading	745	8	2	98.9
Loading to 5 years	735	12	11	97.3
5 years	712	—	—	97.3

CSR: cumulative survival rate.

**Table 7:** Analyses of implant stability at implant insertion.

Variable	Groups	Implants placed	ISQ		Significance test
			Mean	SD	
Age	10–19	7	75.1	6.8	*p*=0.691^(1)^
	20–29	7	75.7	6.7	
	30–39	61	74.2	6.8	
	40–49	164	74.4	7.0	
	50–59	210	72.9	8.9	
	60–69	169	73.2	7.9	
	70–79	33	72.6	6.1	
	80–89	2	77.0	2.8	

Gender	Female	381	72.8	8.1	*p=*0*.002*^(2)^
	Male	264	74.7	7.3	

Jaw	Maxilla	304	71.3	8.4	*p<0.001* ^(2)^
	Mandible	351	75.5	6.8	

Position	Anterior	148	72.3	8.5	*p=*0*.037*^(2)^
	Posterior	506	73.9	7.6	

Indication	Single	142	73.9	8.3	*p=*0*.037*^(1)^ Post hoc test^(3)^ revealed no significant differences between any groups: *p* > 0.022
	Partial	398	74.0	7.2	
	Full	113	71.6	9.2	

Bone quantity	A	25	74.1	7.5	*p*=0.197^(1)^
	B	277	74.0	8.0	
	C	325	73.3	7.5	
	D	27	70.3	10.3	
	E	0	—	—	

Bone quality	1	35	76.5	6.4	*p<0.001* ^(1)^ Post hoc test^(3)^: Bone quality 3 ≠ all other groups: *p* < 0.001 Bone quality 4 ≠ all other groups: *p* < 0.001
	2	354	75.2	7.0	
	3	224	72.0	7.0	
	4	41	63.8	10.7	

Implant diameter	3.5 mm	147	73.1	7.6	*p=*0*.034*^(1)^ Post hoc test^(3)^: 4.0 mm ≠ 4.5 mm: *p*=0.007
	4.0 mm	373	73.4	7.3	
	4.5 mm	117	75.1	8.5	
	5.0 mm	2	66.5	19.1	
	5.5 mm	15	70.9	12.9	

Implant length	7 mm	28	71.0	9.1	*p*=0.187^(1)^
	9 mm	164	73.8	7.3	
	11 mm	186	74.4	6.7	
	13 mm	179	73.8	8.1	
	15 mm	92	71.5	9.4	
	17 mm	5	76.0	6.4	

^(1)^Kruskal–Wallis test; ^(2)^Mann–Whitney *U*-test; ^(3)^pairwise Mann–Whitney *U*-tests (Bonferroni-corrected significance levels).

**Table 8:** Analyses of implant stability at abutment connection or prosthetic loading.

Variable	Groups	Implants	ISQ		Significance test
		Placed	Mean	SD	
Age	10–19	5	80.6	7.2	*p*=0.493^(1)^
	20–29	6	77.5	5.4	
	30–39	45	75.9	5.2	
	40–49	144	74.3	6.8	
	50–59	154	75.2	6.4	
	60–69	140	74.9	7.0	
	70–79	31	76.6	4.4	
	80–89	2	77.0	8.5	

Gender	Female	322	74.7	6.9	*p*=0.075^(2)^
	Male	204	75.9	5.7	

Jaw	Maxilla	245	74.1	6.6	*p=*0*.001*^(2)^
	Mandible	284	76.0	6.3	

Position	Anterior	112	74.8	6.6	*p*=0.533^(2)^
	Posterior	416	75.1	6.5	

Indication	Single	78	74.7	7.9	*p*=0.707^(1)^
	Partial	333	75.0	6.2	
	Full	117	75.7	6.2	

Bone quantity	A	20	76.7	5.9	*p*=0.282^(1)^
	B	210	75.1	6.7	
	C	279	75.1	6.4	
	D	19	73.1	6.5	
	E	0	—	—	

Bone quality	1	23	76.6	5.6	*p=*0*.025*^(1)^ Post hoc test^(3)^: Bone quality 2 ≠ bone quality 3: *p*=0.006
	2	284	75.8	6.5	
	3	183	74.2	6.2	
	4	38	73.5	8.3	

Implant diameter	3.5 mm	106	74.1	6.7	*p=*0*.009*^(1)^ Post hoc test^(3)^: 3.5 mm ≠ 4.5 mm: *p*=0.002 4.0 mm ≠ 4.5 mm: *p*=0.003
	4.0 mm	310	74.8	6.4	
	4.5 mm	100	76.9	6.5	
	5.0 mm	2	77.5	3.5	
	5.5 mm	10	77.8	6.2	

Implant length	7 mm	25	73.5	6.7	*p*=0.585^(1)^
	9 mm	144	75.1	6.9	
	11 mm	151	75.6	5.9	
	13 mm	127	75.0	6.6	
	15 mm	76	75.1	6.6	
	17 mm	5	70.8	7.5	

^(1)^Kruskal–Wallis test; ^(2)^Mann–Whitney *U*-test; ^(3)^pairwise Mann–Whitney *U*-tests (Bonferroni-corrected significance levels).

**Table 9:** Analyses of implant survival.

Variable	Groups	Implants	Implant failures		CSR (%)	Significance test
		Placed	Overall	Early/late		
Age	10–19	7	0	—	100	*p*=0.650^(1)^
	20–29	7	0	—	100	
	30–39	70	2	2/0	97.1	
	40–49	186	2	1/1	98.9	
	50–59	240	9	5/4	96.3	
	60–69	192	7	0/7	96.4	
	70–79	38	0	—	100	
	80–89	3	0	—	100	

Gender	Female	434	13	5/8	97.0	*p*=0.651^(2)^
	Male	301	7	3/4	97.7	

Jaw	Maxilla	368	14	7/7	96.2	*p*=0.071^(2)^
	Mandible	377	6	1/5	98.4	

Position	Anterior	172	7	3/4	95.9	*p*=0.279^(2)^
	Posterior	572	13	5/8	97.7	

Indication	Single	165	5	1/4	97.0	*p=*0*.013*^(1)^ Post hoc test^(3)^: Full ≠ partial: *p*=0.003
	Partial	452	7	5/2	98.5	
	Full	126	8	2/6	93.7	

Bone quantity	A	30	0	—	100	*p*=0.126^(1)^
	B	320	5	4/1	98.4	
	C	365	13	4/9	96.4	
	D	27	2	0/2	92.6	
	E	0	0	—	—	

Bone quality	1	37	0	—	100	*p*=0.740^(1)^
	2	392	11	4/7	97.2	
	3	263	8	4/4	97.0	
	4	50	1	0/1	98.0	

Implant diameter	3.5 mm	155	7	2/5	95.5	*p*=0.431^(1)^
	4.0 mm	447	10	6/4	97.8	
	4.5 mm	122	2	0/2	98.4	
	5.0 mm	3	0	—	100	
	5.5 mm	15	1	0/1	93.3	

Implant length	7 mm	31	1	0/1	96.8	*p*=0.543^(1)^
	9 mm	176	7	2/5	96.0	
	11 mm	202	4	1/3	98.0	
	13 mm	198	7	4/3	96.5	
	15 mm	128	1	1/0	99.2	
	17 mm	7	0	—	100	

ISQ at insertion	<50	7	1	1/0	85.7	*p=*0*.004*^(1)^ Post hoc test^(3)^: 60–69 ≠ 80–89: *p*=0.001
	50–59	32	1	0/1	96.9	
	60–69	112	8	1/7	92.9	
	70–79	346	9	5/4	97.4	
	80–89	158	0	—	100	

ISQ at loading	<50	0	0	0/0	—	*p=*0*.003*^(1)^ Post hoc test^(3)^: 60–69 ≠ 80–89: *p*=0.001
	50–59	6	0	0/0	100	
	60–69	95	7	0/7	92.6	
	70–79	267	4	0/4	98.5	
	80–89	161	1	0/1	99.4	

^(1)^Pearson chi-square; ^(2)^Fisher's exact test; ^(3)^pairwise Fisher's exact tests (Bonferroni-corrected significance levels).

**Table 10:** Implant survival below and above at different threshold values at implant insertion and loading.

Variable	Groups	Implants placed	Implant failures		CSR^(1)^ (%)	Odds ratio	95% C.I.	*p* value
			Overall	Early/late				
ISQ at insertion	<60	39	2	1/1	94.9	1.90	0.4–8.6	0.314^(1)^
	≥60	616	17	6/11	97.2	1		

ISQ at loading	<60	6	0	0/0	100	N/A	N/A	1.0^(1)^
	≥60	523	12	0/12	97.7			

ISQ at insertion	<65	83	5	1/4	94.0	2.56	0.9–7.3	0.080^(1)^
	≥65	572	14	6/8	97.6	1		

ISQ at loading	<65	42	2	0/2	95.2	2.39	0.5–11.2	0.245^(1)^
	≥65	487	10	0/10	97.9	1		

ISQ at insertion	<70	151	10	2/8	93.4	3.90	1.6–9.7	0.004^(1)^
	≥70	504	9	5/4	98.2	1		

ISQ at loading	<70	101	7	0/7	93.1	6.30	1.9–20.2	0.003^(1)^
	≥70	428	5	0/5	98.8	1		

ISQ at insertion	<75	293	16	6/10	94.5	6.90	2.0–23.8	0.001^(1)^
	≥75	362	3	1/2	99.2	1		

ISQ at loading	<75	207	11	0/11	94.7	17.9	2.3–142.9	<0.001^(1)^
	≥75	322	1	0/1	99.7	1		

CSR: cumulative survival rate; ^(1)^Fisher's exact test.

## Data Availability

The data used to support the findings of this study are available from the corresponding author upon request.
